# Liver iron stores and effectors of ferroptosis are dependent on age and sex

**DOI:** 10.1113/EP092035

**Published:** 2024-10-18

**Authors:** Steven A. Bloomer, Brett A. Wagner, Garry R. Buettner, Kyle E. Brown

**Affiliations:** ^1^ Division of Science and Engineering Penn State Abington Abington Pennsylvania USA; ^2^ Free Radical and Radiation Biology, Department of Radiation Oncology University of Iowa Carver College of Medicine Iowa City Iowa USA; ^3^ Iowa City Veterans Administration Medical Center Iowa City Iowa USA; ^4^ Division of Gastroenterology‐Hepatology, Department of Internal Medicine University of Iowa Carver College of Medicine Iowa City Iowa USA

**Keywords:** 4‐hydroxynonenal‐modified proteins, acyl‐CoA synthetase long‐chain family member 4, electron paramagnetic resonance, labile iron

## Abstract

Ferroptosis is a form of cell death characterized by a pro‐oxidative cellular milieu and iron‐dependent lipid peroxidation. Ferroptosis has been implicated in various forms of liver injury, in keeping with the major role of the liver in iron metabolism. Limited research has addressed potential differences in ferroptosis mediators with age and sex, especially in an in vivo model. The goal of this investigation was to evaluate hepatic labile iron and mediators of ferroptosis with ageing in both sexes. Because female animals generally display greater antioxidant defences than males, we hypothesized that females would display a phenotype resistant to ferroptosis. Here, we determined iron contents, protein expression of ferroptosis mediators and measures of oxidative injury in liver samples from 12‐ and 24‐month‐old male and female Fischer 344 rats. In comparison to males, the livers of female rats at both ages contained more non‐haem iron, which was associated with greater ferritin heavy chain expression and attenuated expression of transferrin receptor‐1. In female rats, the 24‐month‐old group had higher contents of thiobarbituric acid reactive substances compared with their 12‐month‐old counterparts, yet similar contents of labile iron. These results suggest a disconnect between labile iron contents and oxidative injury with age. Female animals also displayed greater expression of acyl‐CoA synthetase long‐chain family member 4 (ACSL4), a modulator of ferroptosis, and greater abundance of high molecular weight 4‐hydroxnonenal‐modified proteins. These results demonstrate clear differences in iron and ferroptosis mediators between sexes and suggest that female rats of this strain might be more susceptible to ferroptosis.

## Abstract (highlights)

- **What is the central question of this study?**
  Do iron stores and ferroptosis mediators in the liver differ with age or sex?
- **What is the main finding and its importance?**
  We observed greater non‐haem iron, 4‐hydroxynonenal‐modified proteins and ACSL4 protein expression in female livers, suggesting greater susceptibility to ferroptosis.

## 1 INTRODUCTION

Iron plays a critical role in physiology, demonstrated by its functions in oxygen transport and mitochondrial respiration. In these processes, iron is incorporated either into haem or into iron–sulfur (Fe‐S) centres. The majority of iron not incorporated into haem or Fe‐S centres is stored in the iron storage protein, ferritin. In addition to these forms, cells contain a cytosolic labile iron pool, which consists of ferrous iron, most of which is bound to intracellular glutathione (Hider & Kong, 2013; Philpott & Jadhav, 2019). This iron–glutathione complex is coordinated further by the iron chaperone protein, poly(rC)‐binding protein‐1 (PCBP1; Philpott & Jadhav, 2019; Shi et al., 2008). These multiple layers of iron binding within the cell underscore its potential reactivity. In the presence of reactive oxygen species, such as superoxide or hydrogen peroxide, labile iron catalyses damage to macromolecules such as lipids, proteins and nucleic acids. Accordingly, conditional knockout of the iron chaperone protein, PCBP1, in the liver results in higher levels of catalytically active iron and iron‐mediated lipid peroxidation (Protchenko et al., 2021).

Severe lipid peroxidation owing to oxidative stress, along with high levels of labile iron, cause a necrotic form of cell death termed ferroptosis, which is distinct from apoptosis, yet shares morphological features with necrosis (Dixon et al., 2012). The depletion of ATP in necrosis distinguishes it from ferroptosis (Dixon et al., 2012). Two important protein regulators of ferroptosis are glutathione peroxidase‐4 (GPx4) and acyl‐CoA synthetase long‐chain family member 4 (ACSL4). Using glutathione as a cofactor, GPx4 catalyses the reduction of phospholipid hydroperoxides to the corresponding alcohol; thus, it blunts iron‐mediated lipid peroxidation and inhibits ferroptosis. In HT‐1080 fibrosarcoma cells, inhibition of GPx4 with RAS‐selective lethal compound 3 (RSL3), leads to ferroptotic cell death (Yang et al., 2014). In that study, the addition of the iron chelator deferoxamine to GPx4‐ablated cells suppressed cell death, providing a link between iron and lipid peroxidation in ferroptosis (Yang et al., 2014). ACSL4 activates arachidonic acid (AA) to AA‐CoA and incorporates AA‐CoA into phospholipids, such as phosphatidylethanolamine, which makes this phospholipid a preferred substrate for oxidation (Kagan et al., 2017). Conditional knockout of ACSL4 in TAM‐inducible *Gpx4* ^−/−^ (Pfa1) cells prevents the formation of oxidized AA (Doll et al., 2017). In this cell line and primary mouse hepatocytes (Doll et al., 2017; Grube et al., 2022), ablation of ACSL4 confers protection to RSL3‐mediated ferroptosis; thus, ACSL4 stimulates this cell death pathway.

Ferroptosis has been implicated in a variety of forms of liver pathology (Chen et al., 2022). Sensitivity to ferroptosis is consistent with the role of the liver in whole‐body iron turnover and high levels of constitutive iron flux (Hershko et al., 1973). The liver safely stores excess iron and makes it available when needed (i.e., for erythropoiesis). The primary means of iron uptake is through transferrin receptor‐1 (TFR1), which binds to and internalizes diferric transferrin from the plasma. Iron can then be distributed intracellularly, stored in ferritin or haemosiderin, or be exported from the cell via ferroportin. Several studies have demonstrated differences in hepatic iron and iron regulatory proteins (distinguished here as ferritin and TFR1) between sexes, showing that female rats have more non‐haem iron and more ferritin and less TFR1 expression than male rats (Linder et al., 1973; Ljubojević et al., 2019; Tao et al., 2023; Vulinović et al., 2022). However, less work has been done to evaluate potential sexual dimorphism in mediators of ferroptosis in vivo, particularly in the liver. In one study, sex differences have been compared in the response to iron loading, finding that isolated hepatocytes from female C57BL/6 mice were more resistant to iron‐mediated cell death than male hepatocytes (Tao et al., 2023). In another investigation, it was demonstrated that there was greater overall glutathione peroxidase activity in female rat livers than in male livers (Rikans et al., 1991). In the kidney, a recent investigation has shown that male mice develop greater renal damage and cell death after conditional GPx4 deletion compared with female mice (Ide et al., 2022). These differences were attributed to sexually dimorphic hormone profiles, because ovariectomy sensitized female mice to renal injury after GPx4 knockdown (Ide et al., 2022).

In addition to sex, ageing alters hepatic iron homeostasis. For example, ageing is associated with hepatic iron accumulation in male and female C57BL/6Jrj mice (Lossow et al., 2020) and with ferritin accumulation in male and female Wistar rats (Vulinović et al., 2022). Moreover, liver macrophages in male 24‐month‐old Fischer 344 rats display histologically stainable iron, which suggests iron overload in this cell type. In contrast, male Fischer rats at 6 months of age lack iron deposition in liver macrophages (Bloomer, 2022). Although the causes of iron retention in the liver with ageing remain unclear, age‐related iron accumulation could suggest that the liver is predisposed to ferroptosis. Indeed, 24‐month‐old male C57BL/6J mice have greater hepatic ACSL4 protein, 4‐hydroxynonenal (4‐HNE)‐modified proteins and malondialdehyde than 3‐month‐old mice, which are augmented further in the old mice by a choline‐deficient, high‐fat diet (Du et al., 2024). These elevations in ACSL4 and lipid peroxidation were attenuated by the treatment of animals with ferrostatin‐1, a radical scavenger used to inhibit ferroptosis (Du et al., 2024). These observations were made in male mice only, highlighting the need to compare ferroptosis between sexes. Although we have previously demonstrated similar contents of labile iron between young and old male rats (Bloomer et al., 2008), we have not yet investigated the effect of sex on labile iron. Therefore, the purpose of the present study was to investigate potential sex differences in ferroptosis by comparing labile iron and lipid peroxidation end‐products, in addition to the ferroptosis regulators, GPx4 and ACSL4, in an in vivo ageing model. Based on previous research, we hypothesized that female rats would demonstrate an overall phenotype that would be resistant to ferroptosis compared with their male counterparts at both 12 and 24 months of age.

## 2 MATERIALS AND METHODS

### 2.1 Ethical approval

Frozen livers were provided by the National Institute on Aging (NIA) Aged Rodent Tissue Bank (https://www.nia.nih.gov/research/dab/aged‐rodent‐tissue‐bank) at the University of Washington, Seattle under a contractual agreement with the National Institute on Aging. Animals were kept in the same facility, with the same temperature (20°C–23°C), humidity (40%–70%), 12 h–12 h light–dark cycle and cage enrichment, and were fed sterilized Purina Mills 5L79. Animals were housed three per cage, and the stress of handling animals was minimized. Each animal was euthanized individually by standard carbon dioxide asphyxiation, and tissues were immediately collected, frozen, and stored at −80°C. The frozen livers were sent to the first author's laboratory; therefore, no live animals were housed at the authors’ institutions for this study. Preserved tissues from animals euthanized under an approved Institutional Animal Care and Use Committee (IACUC) protocol are exempt from further IACUC review at the Pennsylvania State University. The experimental groups consisted of 12‐month‐old male (12M, *n* = 6), 12‐month‐old female (12F, *n* = 5), 24‐month‐old male (24M, *n* = 6) and 24‐month‐old female (24F, *n* = 6) Fischer 344 rats. These sample sizes were used for every measure described herein. Power calculations using data from our laboratory and others (Bloomer et al., 2008; Haak et al., 2023; Kozlov et al., 1992; Ljubojević et al., 2019) demonstrated a 90% chance of detecting statistically significant differences at an α level of 0.05 with these sample sizes. The ages chosen represented adult (12‐month‐old) animals; Fischer 344 rats at this age are on the flat part of the survival curve, demonstrating nearly 100% survival (Turturro et al., 1999). At 24 months, survival of this strain declines to ∼60% (Turturro et al., 1999), and the livers of these animals feature the hallmarks of ageing, including augmented oxidative stress (Zhang et al., 2003). Whole livers were ground systematically into small pieces on dry ice for the assays described.

### 2.2 Non‐haem iron

Non‐haem iron was determined using the method of Torrance and Bothwell (1980). Briefly, pieces of frozen liver were thawed, then placed in an oven overnight at 67°C. Dried liver pieces were weighed, and ∼0.01 g was added to acid‐washed glass tubes with 3 mL of acid digest solution (10% trichloroacetic acid in 3 M HCl) and again incubated overnight. Aliquots (800 µL) of the acid extract were combined with 200 µL of freshly prepared chromagen reagent (1 volume of 0.1% bathophenanthroline sulphonate in 0.1% thioglycolic acid added to 10 volumes of 2.5 M sodium acetate) and incubated for 30 min at room temperature. The absorbance of this mixture was read on a spectrophotometer at 532 nm, and absorbance values were compared with a standard curve generated with iron standards (Fisher # Sl 124‐100). All glassware that contained solutions for this assay was acid‐washed with 3 M HCl overnight. Results are expressed as micrograms of iron per gram dry weight of liver.

### 2.3 Immunoblotting

Liver pieces were homogenized in 10 volumes (1.00 mL per 0.10 g tissue) of RIPA buffer (50 mM Tris, pH 7.4, 150 mM NaCl, 0.25% sodium deoxycholate, 1% Triton‐X and 1 mM EDTA) with HALT protease–phosphatase inhibitor added at a 1:100 dilution (Thermo Fisher #87786). The protein content of each lysate was determined using the Bradford protein assay (BioRad #5000006), and lysates were added to 2× Laemmli sample buffer (0.125 M Tris, 4% SDS, 3% 2‐mercaptoethanol, 20% glycerol and 0.004% Bromophenol Blue). We found that boiling the sample completely abolished the 4‐HNE signal; therefore, with the exception of 4‐HNE‐modified proteins, samples were boiled for 5 min before electrophoresis. Equal amounts of protein (66 µg) were loaded onto freshly poured 12% polyacrylamide gels and run at 100 V for ∼90 min. The protein gels were then transferred to nitrocellulose membranes at 100 V for 90 min. Then, membranes were blocked in a solution of 5% non‐fat dry milk and Tris‐buffered saline with tween (TBST) for 30 min. Primary antibody incubations for haem oxygenase‐1 (HO‐1), ACSL4, complex IV (Comp IV) and manganese superoxide dismutase (MnSOD) were carried out for 45 min at room temperature, whereas incubations for ferritin heavy chain (FTH), transferrin receptor‐1 (TFR1), GPx4, thioredoxin‐2 (Trx2) and 4‐HNE‐modified proteins were done overnight at 4°C. Table 1 provides dilutions and sources for each antibody. After primary incubation, membranes were washed three times in TBST, then incubated for 45 min at room temperature with the appropriate secondary antibodies: sheep anti‐mouse‐ECL (Amersham, NA931V, 1:4000) for ACSL4, Comp IV, HO‐1, GPx4 and TFR1, or goat anti‐rabbit‐ECL (Santa Cruz, sc‐2030, 1:4000) for MnSOD, FTH, Trx2 and 4‐HNE. Membranes were washed three times in TBST for 5 min each and treated with Super Signal West Pico Plus chemiluminescent reagent (Thermo Fisher, #34577) for 5 min. Membranes were placed in the Chemi‐Doc XRS+ imager (BioRad) and photographed. Comparisons between only two groups were performed on the same gel (e.g., 12M vs. 12F) with five or six samples per group. Immunoblotting analysis was done in this manner until all four relevant comparisons were made (12M vs. 12F, 12M vs. 24M, 12F vs. 24F, and 24M vs. 24F). Membranes were washed with water, then treated with Ponceau S solution (0.2% Ponceau S, Acros Organics, in 5% acetic acid) for 5 min and then rinsed with water to remove the background. Each protein of interest was normalized to the density of the Ponceau stain. All groups were normalized to the 12M group, which was given a value of one. After these data were generated, we performed blotting on two representative animals from each group; these images are shown in the figures. The full membranes of each blot used for representative images are shown in the Supporting Information.

**TABLE 1:** Antibody suppliers and dilutions.

Antigen	Company	Catalogue number	Dilution
Ferritin	Santa Cruz	sc‐25617	1:500
TFR1	Invitrogen	136800	1:500
HO‐1	Enzo	ADI OSA 111‐F	1:1000
Trx2	Cell Signaling	13322	1:500
Comp IV	Thermo Fisher	A21348	1:4000
MnSOD	Enzo	ADI‐SOD‐110	1:4000
ACSL4	Santa Cruz	sc‐365230	1:500
GPx4	Santa Cruz	sc‐166570	1:500
4‐HNE	Stressmarq	SMC‐511	1:500

### 2.4 Hepatic labile iron

Frozen liver pieces (0.2 g) were homogenized in 400 µL of PBS, pH 6.5 with 1 mM deferoxamine (Kozlov et al., 1992; Moser et al., 2013). Aliquots (200 µL) of each lysate were transferred to a 4 mm o.d. glass EPR tube (Wilmad‐LabGlass, 707‐SQ‐250M) and subsequently frozen in liquid nitrogen. Labile iron was determined via electron paramagnetic resonance (EPR; Bruker EMX EPR spectrometer) using the signal height of the ferrioxamine (Fe^3+^‐DFO) signal at *g* = 4.3 at 100 K using instrument parameters: centre field 1575 G, sweep width 500 G, microwave frequency 9.766 GHz, power 20 mW, receiver gain 2 × 10^5^, modulation frequency 100 kHz, modulation amplitude 2 G, time constant 163.84 ms, conversion time 20.48 ms, and resolution 1024 points. Each spectrum is a result of five additive scans; after the collection of a spectrum, samples were repositioned within the EPR cavity, and a new spectrum was collected. This process was repeated three times. The average signal height of each sample was then used to determine the content of labile iron by comparing it with a standard curve generated by adding increasing contents of FeSO_4_ (5, 10, 15 and 20 µM) to a separate liver sample in the presence of 1 mM deferoxamine (DFO). DFO drives the oxidation of Fe^2+^ to Fe^3+^, hence the formation of ferrioxamine as a standard. Iron contents are expressed as micromoles of Fe per kilogram of tissue wet weight.

### 2.5 Thiobarbituric acid reactive substances

Frozen liver pieces were homogenized in 10 volumes of 20 mM Tris, pH 7.4 with 1.34 mM diethylenetriaminepentaacetic acid (DETAPAC; Sigma #D6518), and 5 mM butylated hydroxytoluene (BHT; Sigma W218405). Aliquots (500 µL) were treated with 250 µL 15% trichloroacetic acid for 5 min, then centrifuged at 3000*g* for 10 min. Supernatants (500 µL) were treated with 250 µL of 0.7% thiobarbituric acid in 50% acetic acid, vortexed, then boiled for 30 min. Samples were cooled on ice and read in a spectrophotometer at 532 nm. Absorbances of the samples were compared with a standard curve generated with tetraethoxypropane and normalized to the protein content of each sample. Results are expressed as nanomoles of thiobarbituric acid reactive substances (TBARS) per milligram of protein.

### 2.6 Statistics

All values for a given parameter (e.g., non‐haem iron), within each sample set (12M, 12F, 24M and 24F) were checked for normality with a Shapiro–Wilk test prior to statistical tests. The only data set that was not normally distributed was the 12F group for ‘All’ bands of 4‐HNE‐modified proteins; therefore, comparisons including this group were performed using a non‐parametric test (Mann–Whitney *U*‐test). The significance of every other measure was determined with a two‐factor ANOVA, with age and sex as the two main factors. *Post hoc* Student's *t*‐tests for independent samples were performed between groups to determine significance only when the ANOVA first indicated significant (*P* < 0.05) main effects of age, sex, or both. To minimize the risk of a type I error, we controlled for multiple comparisons using a Bonferroni correction (0.05 divided by the number of comparisons). When both main factors of age and sex were significant, there were four *post hoc* comparisons (12M vs. 24M, 12F vs. 24F, 12M vs. 12F, and 24M vs. 24F), and we used an adjusted *P*‐value of 0.0125. When only one main factor (e.g., sex) was significant, there were only two *post hoc* comparisons (12M vs. 12F and 24M vs. 24F); therefore, an adjusted *P*‐value of 0.025 was used.

## 3 RESULTS

### 3.1 Non‐haem iron and iron‐regulatory proteins

**FIGURE 1:**
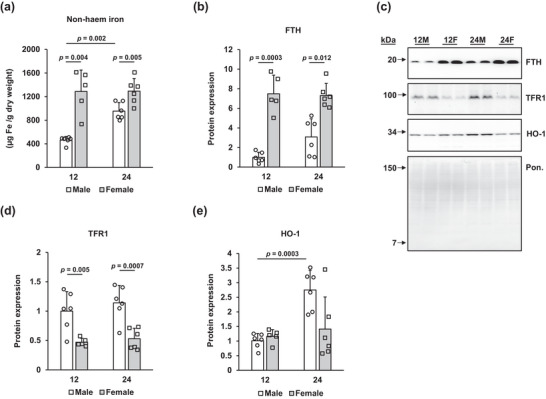
Greater non‐haem iron and ferritin in female animals. (a) Non‐haem iron was determined spectrophotometrically and expressed as the mean + SD in units of micrograms of iron per gram dry weight of liver. (b, d, e) Ferritin(b), transferrin receptor‐1 (TFR1; d) and haem oxygenase‐1 (HO‐1; e) were determined by western blot, and band brightness was normalized to the density of the Ponceau stain. (c) Representative blots are shown, with the Ponceau‐stained membrane underneath. The representative Ponceau blot was probed for HO‐1. All results are expressed as the mean + SD, with individual values shown. Values indicating significance are shown; only values that reached significance are reported. Sample sizes are as follows: 12‐month‐old male (12M), *n* = 6; 12‐month‐old female (12F), *n* = 5; 24‐month‐old male (24M), *n* = 6; and 24‐month‐old female (24F), *n* = 6. FTH: ferritin heavy chain; HO‐1: haem oxygenase‐1; TFR1: transferrin receptor‐1.

Significant main effects of age (*P* = 0.013) and sex (*P* < 0.001) were determined for non‐haem iron. Female animals at 12 months of age demonstrated higher non‐haem iron contents than their male counterparts (*P* = 0.004); this sex difference was also observed in the 24‐month‐old cohort (*P* = 0.005; Figure 1a). Although non‐haem iron did not change with age in female rats, it increased significantly between 12 and 24 months in male rats (*P* = 0.002). These sex differences in non‐haem iron paralleled ferritin protein expression, for which we observed a significant main effect of sex only (*P* < 0.001). Female animals at 12 months demonstrated a ∼7‐fold increase in hepatic ferritin expression compared with males of the same age (*P* = 0.0003; Figure 1b). At 24 months, ferritin expression in the livers of female animals was still more than twice that of their male counterparts (*P* = 0.012). We determined a significant main effect of sex only on transferrin receptor 1 (TFR1) expression (*P* < 0.001). The differences in TFR1 were the inverse of those of ferritin, with males at 12 (*P* = 0.005) and 24 months (*P* = 0.0007) having greater expression of TFR1 than aged‐matched females (Figure 1d). Finally, given that HO‐1 degrades haem and produces labile iron, which is then incorporated into ferritin, we characterized protein levels of HO‐1. Two‐factor ANOVA revealed a significant main effect of ageing (*P* = 0.003), on HO‐1 with 24‐month‐old males having higher levels than 12‐month‐old males (*P* = 0.0003; Figure 1e).

### 3.2 Labile iron

**FIGURE 2:**
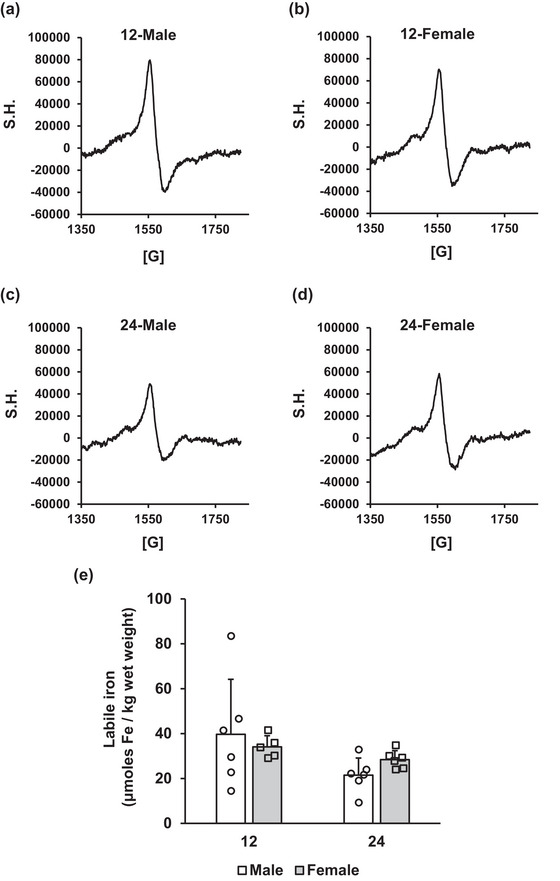
Hepatic labile iron in male and female rats. Liver samples were tested for labile iron contents via electron paramagnetic resonance spectroscopy (EPR). Representative EPR spectra of the iron (III) deferoxamine complex are shown for 12M (a), 12F (b), 24M (c) and 24F (d) animals. (e) Results are quantified and expressed as the mean + SD in units of micromoles of iron per kilogram wet weight. Sample sizes are as follows: 12‐month‐old male (12M), *n* = 6; 12‐month‐old female (12F), *n* = 5; 24‐month‐old male (24M), *n* = 6; and 24‐month‐old female (24F), *n* = 6. S.H. is signal height. [G] is magnetic field.

The levels of labile iron determined in this study are within the SD for mouse liver as described previously (Moser et al., 2013) and closely approximate values observed by Kozlov et al. (1992) in the rat liver. We observed a significant main effect of ageing (*P* = 0.048) on EPR‐detectable iron in the liver, which approached statistical significance in the female animals only, with 24‐month‐old animals having lower labile iron than the 12‐month‐old group (*P* = 0.032; Figure 2). In male rats, 24‐month‐old animals tended to have lower labile iron (*P* = 0.056). There were no significant effects of sex on labile iron.

### 3.3 Regulators of ferroptosis and mitochondrial proteins

**FIGURE 3:**
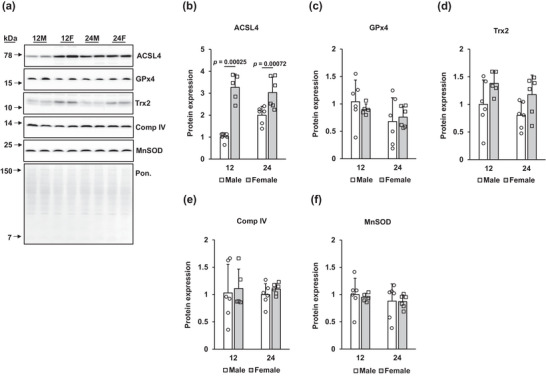
Expression of protein mediators of ferroptosis and mitochondrial proteins. (a) Representative blots are shown. The representative Ponceau blot was probed for MnSOD. (b–f) Protein expression of ACSL4 (b), GPx4 (c), Trx2 (d), Comp IV (e) and MnSOD (f), normalized to the Ponceau density. Results are expressed as the mean + SD, with individual values shown. Values indicating significance are shown. Sample sizes are as follows: 12‐month‐old male (12M), *n* = 6; 12‐month‐old female (12F), *n* = 5; 24‐month‐old male (24M), *n* = 6; and 24‐month‐old female (24F), *n* = 6. ACSL4: acyl‐CoA synthetase long‐chain family member 4; Comp IV: complex 4; GPx4: glutathione peroxidase 4; MnSOD: manganese superoxide dismutase; Trx2: thioredoxin 2.

A significant main effect of sex was observed on ACSL4 protein (*P* < 0.001); female rats at 12 and 24 months of age had higher ACSL4 protein expression than their male counterparts (*P* = 0.00025 and 0.00072, respectively; Figure 3a,b). We did not observe significant changes in GPx4 protein with age or sex (Figure 3c).

Given the importance of the cellular redox environment in ferroptosis, we were interested in determining the expression of important mitochondrial proteins. We determined a significant main effect of sex (*P* = 0.013) on thioredoxin‐2 (Trx2) expression, with greater expression in female rats (Figure 3d). The 12‐ and 24‐month‐old females tended to have higher Trx2 expression than their age‐matched male counterparts (*P* = 0.055 and *P* = 0.029, respectively). Protein expression of the electron transport chain complex IV (Comp IV) and manganese superoxide dismutase (MnSOD) did not differ between age groups or sex in whole liver lysates (Figure 3e,f).

### 3.4 Lipid peroxidation and oxidative injury

**FIGURE 4:**
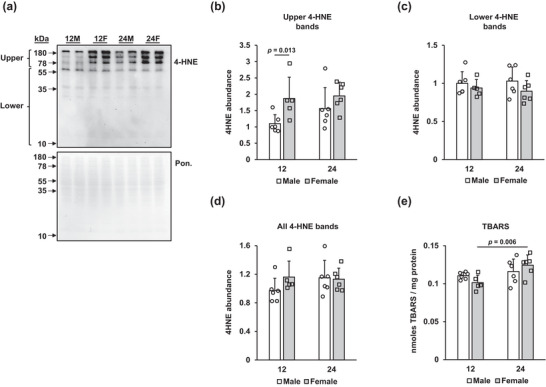
Female sex increases 4‐hydroxynonenal (4‐HNE)‐modified proteins. The abundance of 4‐HNE‐modified protein was detected by immunoblot (a), normalized to the Ponceau stain and grouped as upper molecular weight bands (b; 78–180 kDa), lower molecular weight bands (c; 10–77 kDa) or all bands (d; 10–180 kDa). (e) Thiobarbituric acid reactive substances (TBARS) were determined spectrophotometrically and expressed as nanomoles of TBARS per milligram of protein. Results are expressed as the mean + SD, with individual values shown. Values indicating significance are shown; only values that reached significance are reported. Sample sizes are as follows: 12‐month‐old male (12M), *n* = 6; 12‐month‐old female (12F), *n* = 5; 24‐month‐old male (24M), *n* = 6; and 24‐month‐old female (24F), *n* = 6.

Detection of proteins modified by lipid peroxidation products, such as 4‐HNE, via immunoblotting or immunohistochemistry is a common way to detect a marker of ferroptosis (Liu et al., 2020). At relatively high molecular weights (78–180 kDa), our immunoblots demonstrated differences in signal intensity between groups; therefore, we quantified all immunoreactive bands within the 78–180 kDa range together (‘Upper bands’) and the bands within the 10–77 kDa range together (‘Lower bands’). Additionally, the densities of bands within the entire lane of each sample were quantified (‘All 4‐HNE bands’; Figure 4). Within the upper bands, the two‐factor ANOVA revealed a significant main effect of sex, with females having more 4‐HNE (*P* = 0.014). *Post hoc* analysis of the upper 4‐HNE bands demonstrated that the 12‐month‐old female rats had more 4‐HNE compared with their 12‐month‐old male counterparts (*P* = 0.013; Figure 4a,b). However, when comparing the entire lanes, or only the lower bands, there were no significant differences between sexes (Figure 4a,c,d). We did not observe significant effects of age on 4‐HNE‐modified proteins.

Although the TBARS assay is commonly used as a measure of lipid peroxidation in biological samples, we note that other oxidized macromolecules can react with thiobarbituric acid; thus, the assay is less specific to lipid peroxidation than other assays, such as the measurement of F2‐isoprostanes (Forman et al., 2015; Janero, 1990). However, because contents of liver TBARS increase after treatments that induce oxidative stress, such as severe iron overload, paraquat and carbon tetrachloride (Bloomer & Brown, 2015; Mitchell et al., 2009; Semeniuk et al., 2021), here we consider the assay to be a useful marker of generalized oxidative injury. We found a significant main effect of ageing in increasing TBARS contents (*P* = 0.01) that reached significance in the 24‐month‐old female group only, in comparison to their 12‐month‐old counterparts (*P* = 0.006; Figure 4e).

## 4 DISCUSSION

In this investigation, we have demonstrated clear effects of age and sex on liver iron stores, iron regulatory proteins and mediators of ferroptosis. Specifically, in comparison to male rats, female animals had more non‐haem iron and ferritin and lower expression of TFR1. Combined, these results demonstrate a physiologically appropriate response to higher non‐haem iron contents. In female livers, we demonstrated higher ACSL4 protein and 4‐HNE‐modified protein expression than in males. Furthermore, with the exception of 4‐HNE‐modified proteins, these sex differences in iron regulatory proteins and ACSL4 were maintained into senescence. These results suggest that female animals are predisposed to ferroptosis. However, Tao et al. (2023) have demonstrated less cell death and mitochondrial reactive oxygen species production after iron loading in hepatocytes isolated from female compared with male C57BL/6 mice. Their results could be attributable to higher baseline expression of ferritin, less expression of mitoferrin‐1 (Mfrn1; a mitochondrial iron importer) and/or a greater decline in transferrin receptor‐1 protein in female cells after iron loading (Tao et al., 2023). In addition to iron regulatory proteins, greater antioxidant protein activity or expression in female rats might confer protection from ferroptosis, despite higher ACLS4 expression. For example, although Tao et al. (2023) observed similar protein levels of GPx4 in males and females, an early investigation demonstrated that female rats had greater overall glutathione peroxidase activity in their livers than male rats (Rikans et al., 1991). These studies emphasize the need to compare sex differences in the effects of iron loading on ferroptosis in an in vivo model.

Our results showing greater expression of ACSL4 in female rats are consistent with the effects of sex hormones on ACSL4. In two breast cancer cell lines (MCF‐7 and T47D cells), estradiol augmented protein expression of ACSL4 by preventing its degradation via the proteasome (Belkaid et al., 2017). Conversely, in rat penile cavernous endothelial cells (CP‐R133 cells), dihydrotestosterone decreased ACSL4 protein expression (Shi et al., 2023). We are unaware of studies showing hormonal influences on ACSL4 expression in the liver; thus, this represents an area of future investigation. In the study by Shi et al. (2023), the authors also found that dihydrotestosterone induced GPx4 in CP‐R133 cells. Likewise, the treatment of male Wistar rats with dehydroepiandrosterone increased GPx4 in the epididymis (Tatara & Sato, 2019). We and others (Tao et al., 2023) did not observe differences in liver GPx4 expression between male and female rats, suggesting tissue‐specific differences in the effects of sex hormones on this protein.

Relatively few investigations have measured changes in the labile iron pool with ageing. In the present study, we observed similar contents of labile iron in each group. Given that oxidative injury (TBARS) was greater in 24‐ compared with 12‐month‐old female rats, these results might seem paradoxical. However, other pathways that are independent of iron can lead to lipid peroxidation. For example, treatment of cells with hydrogen peroxide causes oxidative injury, even in the presence of deferoxamine, an iron chelator (Chen et al., 2023). Furthermore, in a previous study, we observed no significant age‐related difference in hepatic labile iron but found that old male rats had greater contents of the lipid peroxidation product malondialdehyde than young rats (Bloomer et al., 2008). Thus, levels of oxidative damage are not always correlated directly with labile iron. It is also possible that age‐related changes in labile iron vary among organs and even cell types. For example, Doulias et al. (2008) demonstrated an age‐related increase in the labile iron pool in human leucocytes. Also, Picca et al. (2020) found evidence of more labile iron in skeletal muscle from older humans (∼80 years of age) compared with younger humans (22 years of age). Therefore, a comprehensive investigation of changes in the labile iron pool with ageing in multiple organs is warranted.

Although labile iron contents were similar between age groups, 24‐month‐old male livers contained >2‐fold more non‐haem iron than their 12‐month‐old counterparts. This difference between age groups has been observed previously in our laboratory, albeit between 6‐ and 24‐month‐old male rats (Bloomer et al., 2008). Interestingly, despite the elevation in non‐haem iron in the older male rats, TFR1 levels were similar between age groups. This could represent the competing effects of iron, which would lower TFR1, and chronic inflammation, which would augment TFR1 protein (Naz et al., 2012; Sukumaran et al., 2012). In the male rats, we also observed higher HO‐1 protein levels in the 24‐month‐old group compared with their 12‐month‐old counterparts. With age, there is an increase in the number of liver macrophages (Bloomer, 2022), which express HO‐1 constitutively; therefore, the higher numbers of macrophages could contribute to this observation. Additionally, this increase in HO‐1 could be attributable to its exquisite sensitivity to cellular stressors that accrue with ageing, such as endotoxin and oxidative stress (Bauer et al., 1998).

Ours is one of several studies to have demonstrated higher levels of liver non‐haem (storage) iron in female rodents versus males (Bowering & Norton, 1981; Hershko & Eilon, 1974; Kong et al., 2014), but to our knowledge, it is the first study to have compared hepatic labile iron between the sexes. Despite the higher levels of non‐haem iron in female rat livers in both age groups, contents of labile iron were similar in males and females. These results suggest a greater flux of iron into storage in the female rats, which is consistent with an elegant paper using isotopic iron to study sex differences in body iron stores. In that study, Hershko and Eilon (1974) demonstrated more liver iron and serum iron in female compared with male Wistar rats. The greater amount of iron in female rats was not attributable to less hepatic iron efflux owing to lower erythropoiesis but to greater dietary uptake of iron and subsequent hepatic iron loading (Hershko & Eilon, 1974).

To determine markers of ferroptosis, we measured TBARS, in addition to proteins modified with the lipid peroxidation end‐product, 4‐HNE. In keeping with earlier and more contemporary studies on hepatic TBARS in rats, we observed similar amounts between the sexes (Chen et al., 2019; Sato et al., 1983). However, some studies have measured greater TBARS in hepatic samples from female mice (Sobočanec et al., 2008) and rats (Ljubojević et al., 2019) compared with males. In the latter two studies, it is unclear whether the authors included an iron chelator, such as Diethylenetriaminepentaacetic acid (DETAPAC), in the TBARS reaction. Without iron chelation, endogenous iron can augment TBARS production during the assay procedure itself (Bloomer et al., 2020); therefore, the greater content of TBARS in the females in those studies could be an artefact of greater endogenous iron content. With respect to age, we demonstrated the main effect of ageing in 24‐month‐old female animals, which have greater TBARS than their 12‐month‐old counterparts. Our analysis of 4‐HNE‐modified proteins demonstrated that 12‐month‐old female rats had a greater number of proteins with 4‐HNE adducts at relatively high molecular weights, in comparison to 12‐month‐old males. These sex differences in a subset of 4‐HNE modified proteins is an observation that, to our knowledge, has not yet been described. Interestingly, the molecular weight of 4‐HNE is 156 kDa, and dehydrated 4‐HNE weighs 138 kDa (Carini et al., 2004); therefore, 4‐HNE adduction can result in protein molecular weight modifications of 156 or 138 kDa if the protein undergoes Michael or Schiff base adduction, respectively (Carini et al., 2004). Also, 4‐HNE modifies enzymes such as glucose‐6‐phosphate dehydrogenase (79 kDa), cathepsin B (38 kDa), glyceraldehyde‐3‐phosphate dehydrogenase (36 kDa) and cytochrome oxidase (12 kDa; Carini et al., 2004), which are abundant in the liver. Modification with 4‐HNE would result in the detection of 4‐HNE‐modified proteins within a molecular weight range of 150–235 kDa, which fits within the range we observed. Future studies are needed to determine sex‐related differences in the specific proteins that are modified by 4‐HNE.

In summary, our results demonstrate that the female sex is associated with greater liver storage of iron and appropriate compensatory changes in iron‐regulatory proteins, in addition to greater expression of the ferroptosis stimulator, ACSL4, and an augmentation in 4‐HNE‐modified proteins. The similar contents of labile iron between males and females of both ages suggest that labile iron contents do not predict sex‐ or age‐related changes in oxidative injury in this model. This observation is likely to be attributable to the capacity of the liver to limit toxic iron through storage in ferritin and other intracellular chelators. Given that ferroptosis is implicated in liver pathologies (Chen et al., 2022) and that some liver diseases display sexual dimorphism (Kasarinaite et al., 2023), our investigation has demonstrated that sex differences must be borne in mind when evaluating ferroptosis in the liver.

## AUTHOR CONTRIBUTIONS

With the exception of the labile iron experiments, all other experiments were performed in the laboratory of Dr Steven A. Bloomer at Penn State Abington. The EPR experiments were performed in the Electron Spin Resonance facility at the University of Iowa. Steven A. Bloomer conceived and designed the work, acquired and analysed the data, drafted the manuscript and made revisions. Brett A. Wagner assisted in acquiring the data and revised the work critically for important intellectual content. Garry R. Buettner assisted in the study design and data interpretation, and critically revised the manuscript. Kyle E. Brown assisted in the study design and data interpretation, and critically revised the manuscript. All authors approved the final version of the manuscript and agree to be accountable for all aspects of the work in ensuring that questions related to the accuracy or integrity of any part of the work are appropriately investigated and resolved. All persons designated as authors qualify for authorship, and all those who qualify for authorship are listed.

## CONFLICT OF INTEREST

None declared.

## Supporting information



Supporting Information

## Data Availability

Data are available from the corresponding author upon reasonable request.
