# Supplementary material for: Liver iron stores and effectors of ferroptosis are dependent on age and sex
Source: Exp Physiol. 2024 Oct 18;109(12):2046–56. doi: 10.1113/EP092035 (PMC11607622; doi:10.1113/EP092035)

Ferritin heavy chain (FTH)

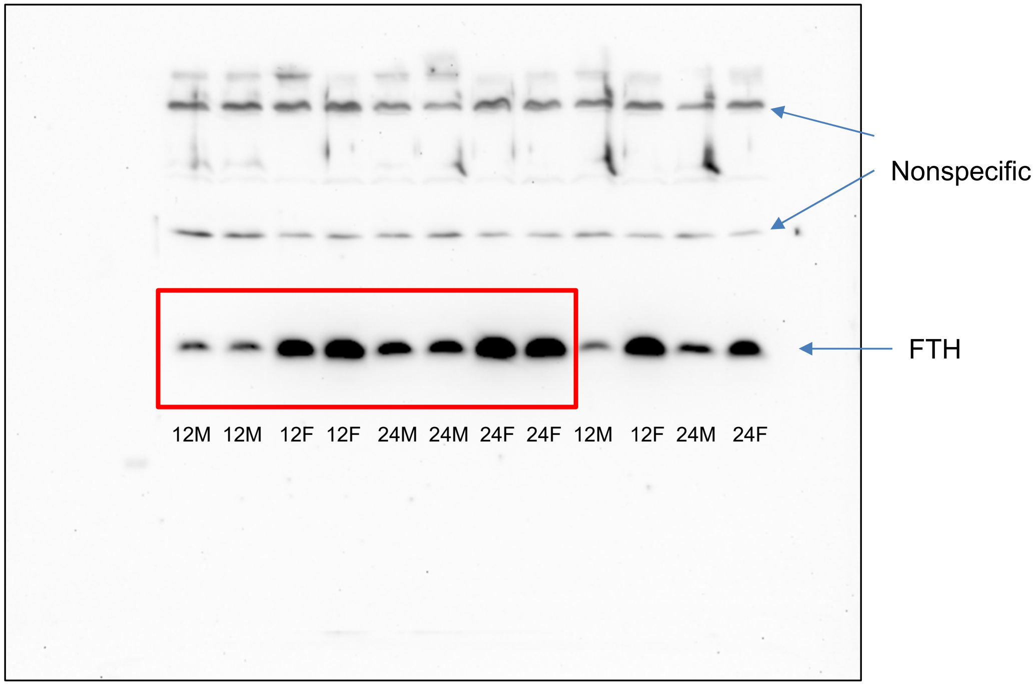

Section used for representative blot is boxed in red.

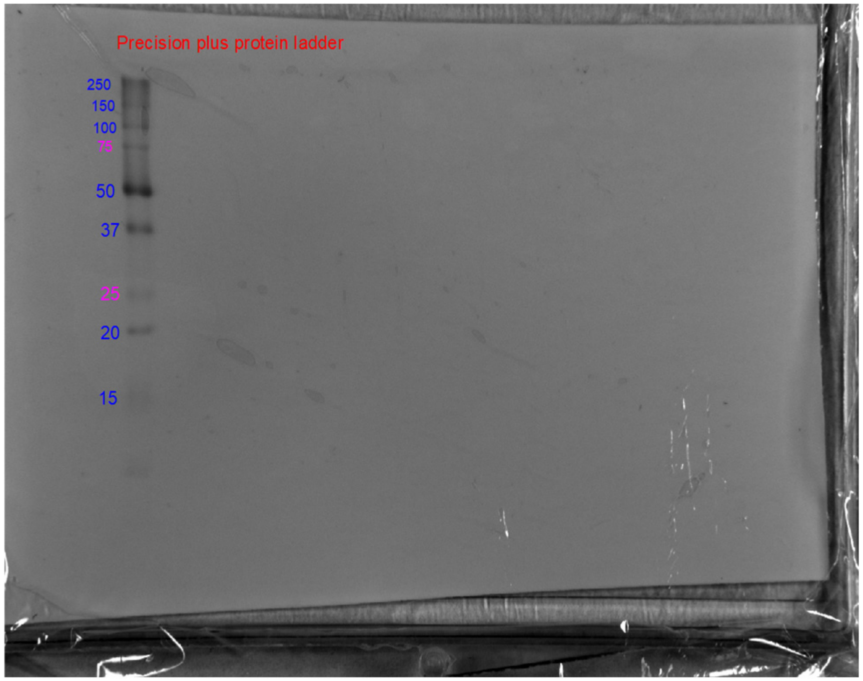

Brightfield

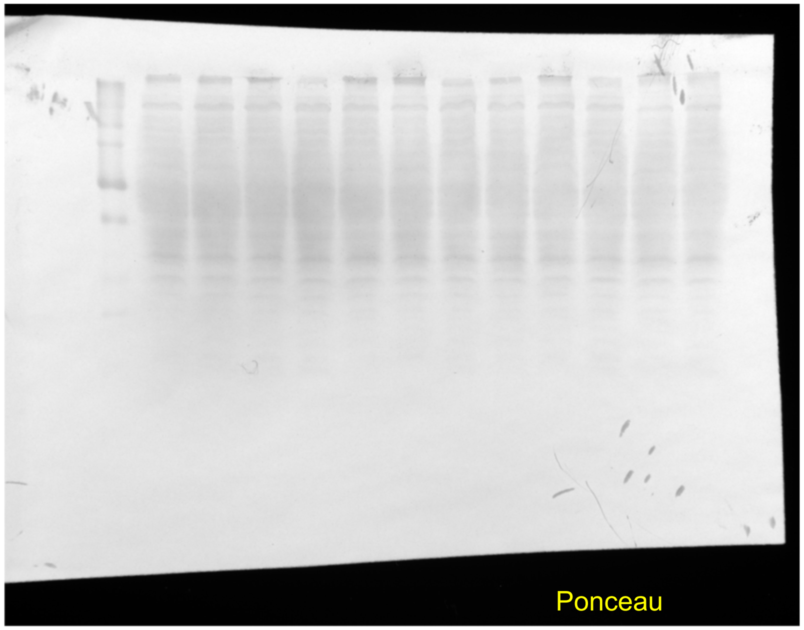

Transferrin receptor -1 (TFR1)

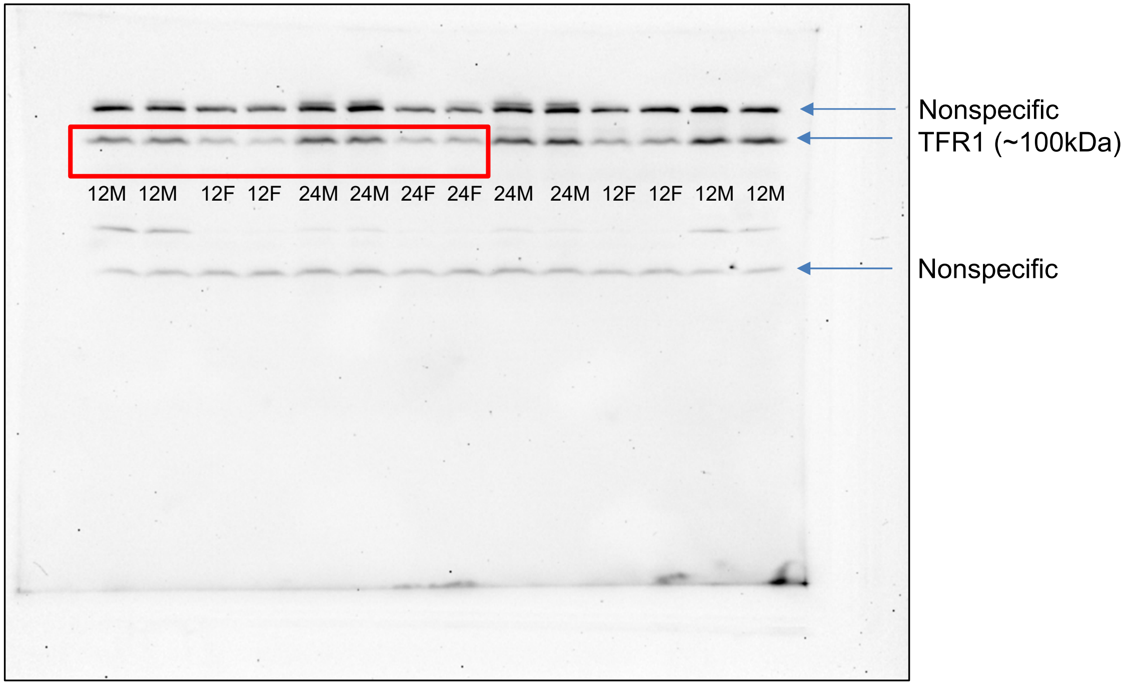

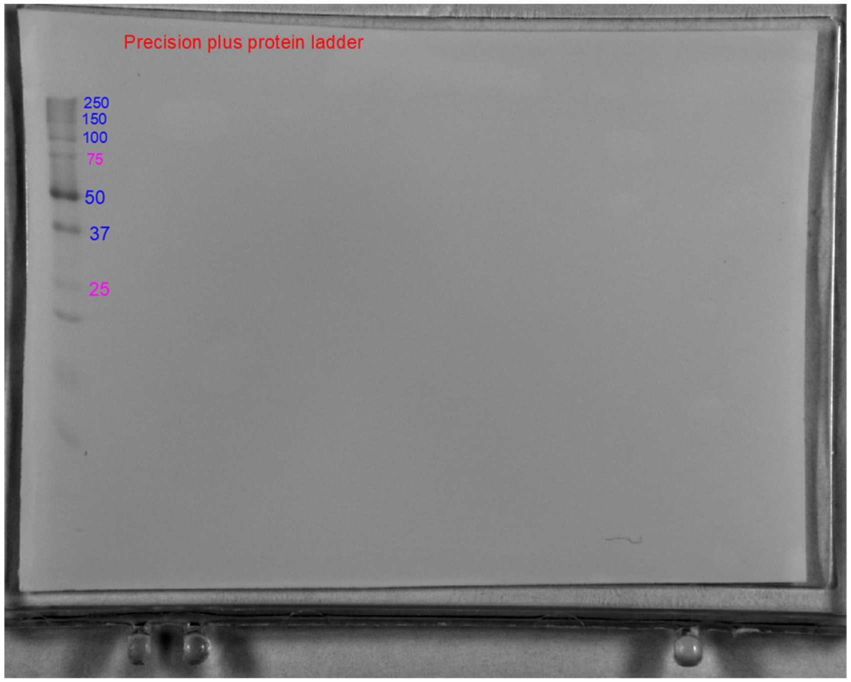

Section used for representative blot is boxed in red.

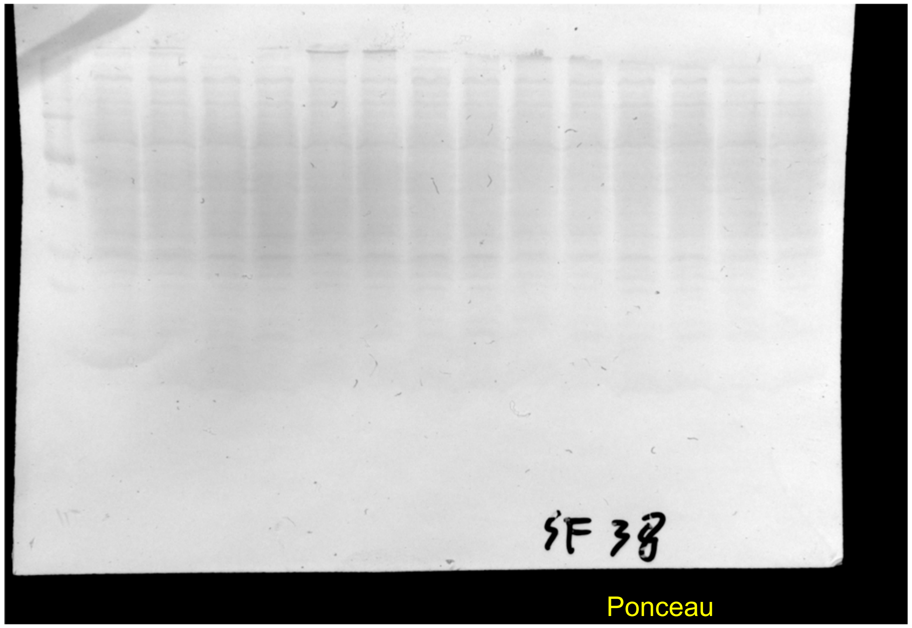

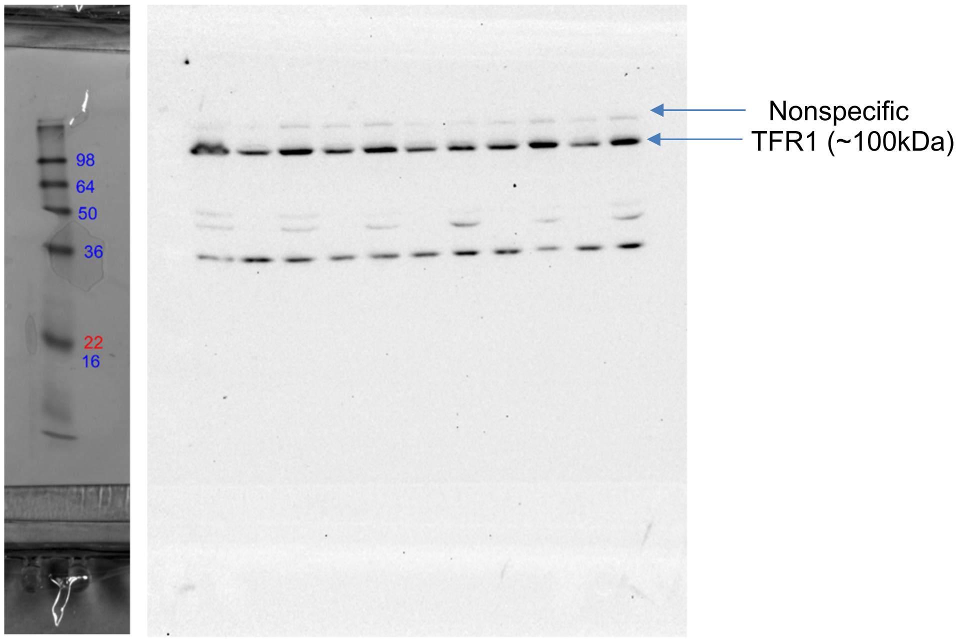

Separate blot (used for data) confirming that higher bands are nonspecific

Heme oxygenase-1 (HO1)

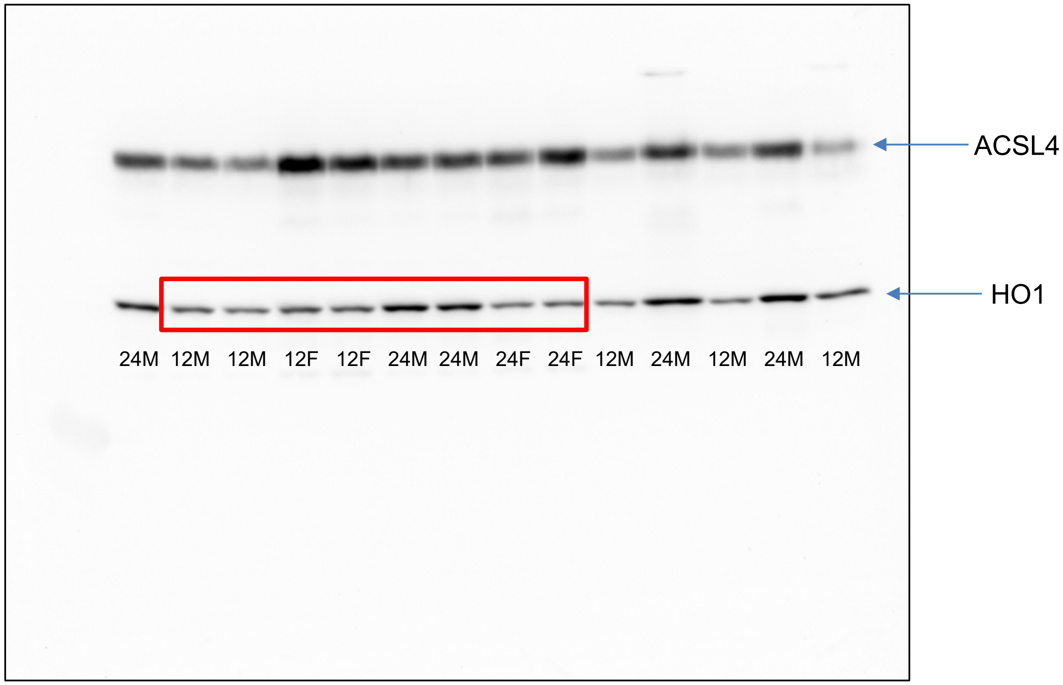

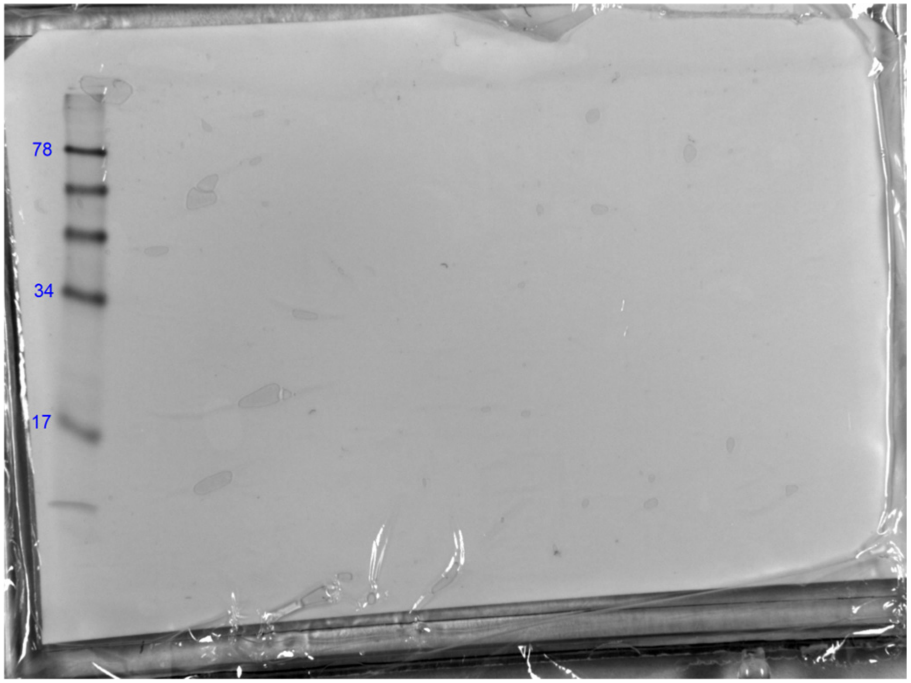

Brightfield

Sections used for representative blots are boxed in red. Blot was probed simultaneously for ACSL4 and HO1.
Extra samples were run for data and to ensure that representative samples were not on the edges of the gel.

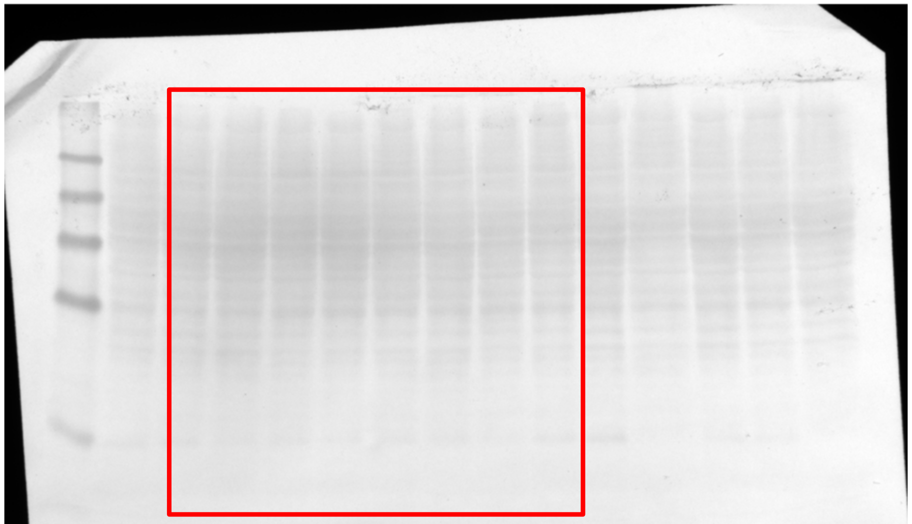

Representative Ponceau for Fig 1.

(ACSL4 and HO-1 were probed simultaneously on the same representative blot.
Insets below show that they gave a single band when probed individually)

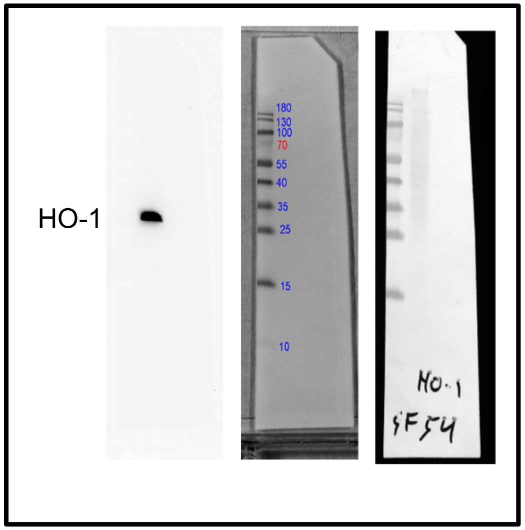

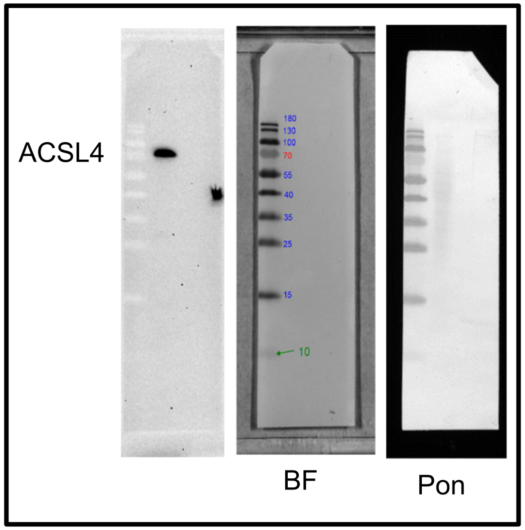

ACSL4

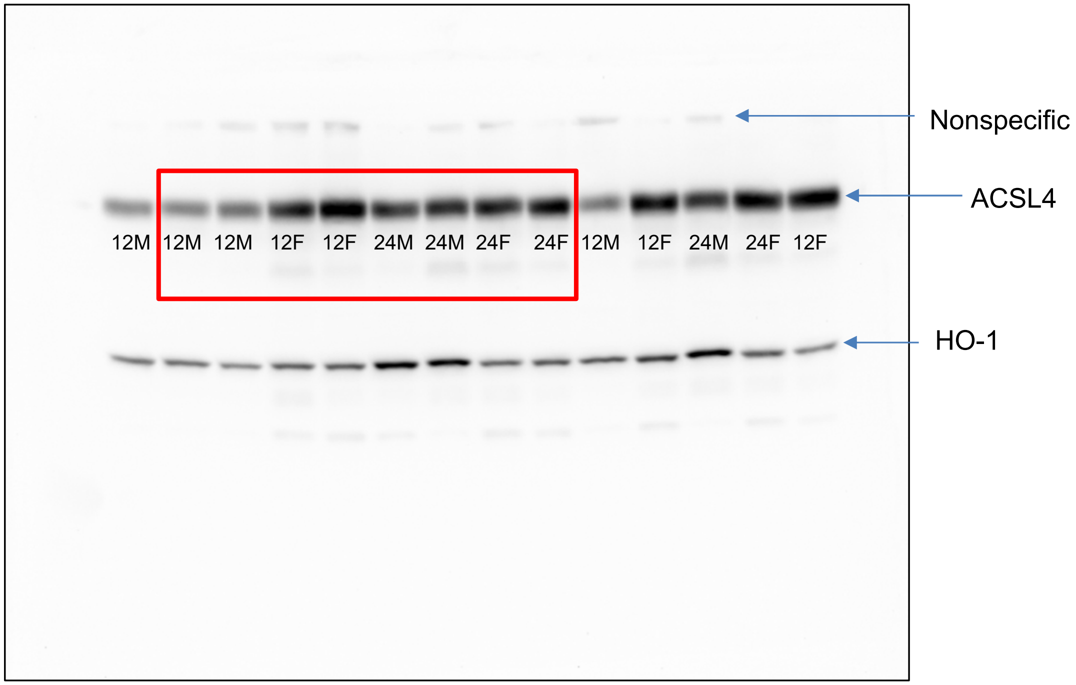

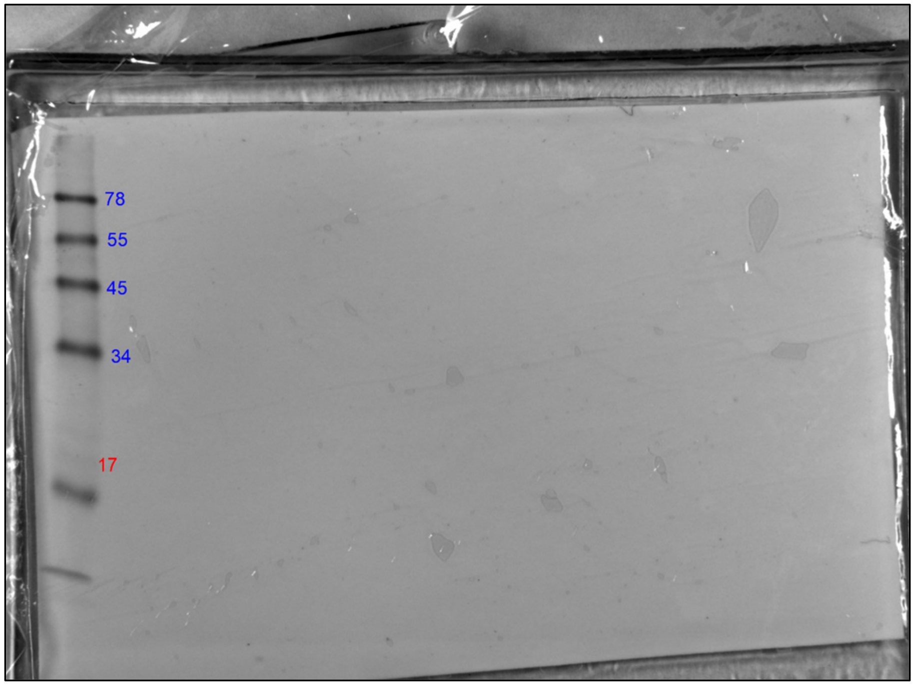

Brightfield

Sections used for representative blots are boxed in red.
Extra samples were run for data and to ensure that representative samples were not on the edges of the gel.

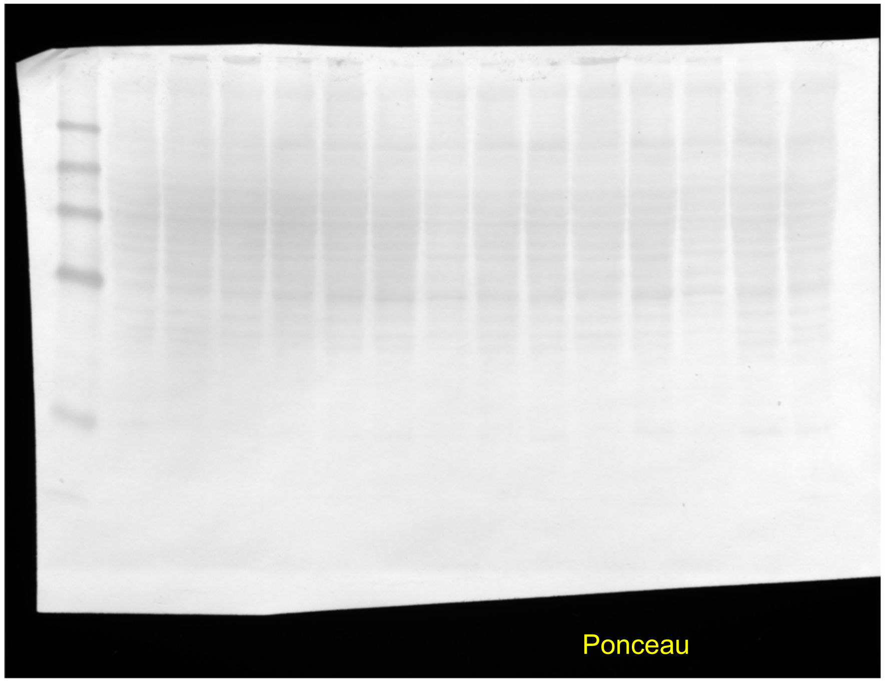

Glutathione peroxidase-4 (GPx4)

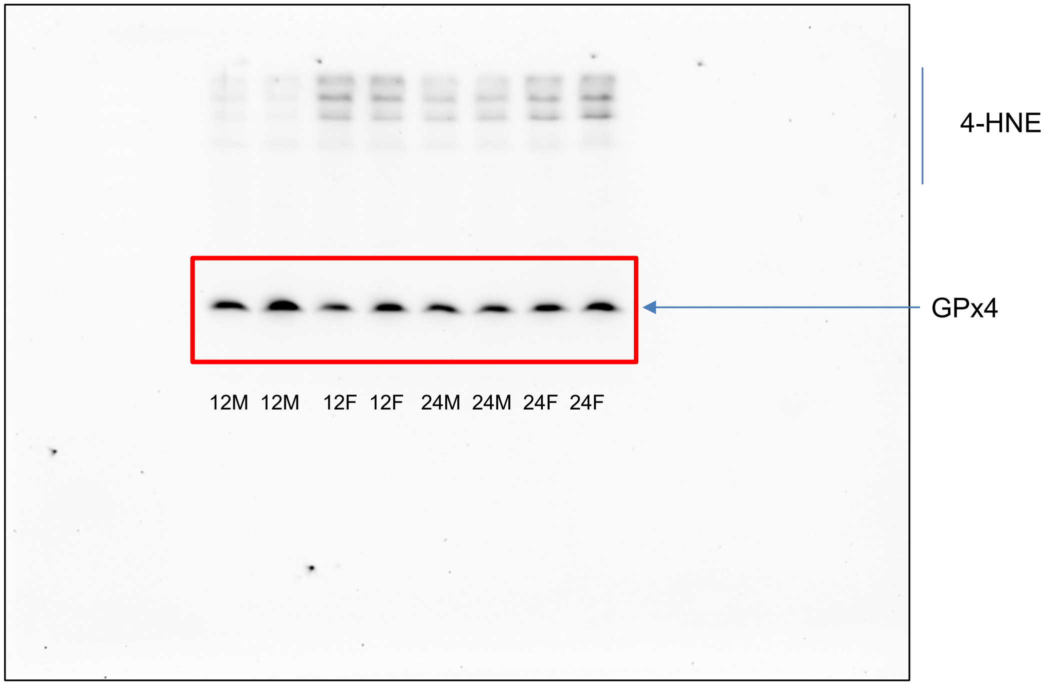

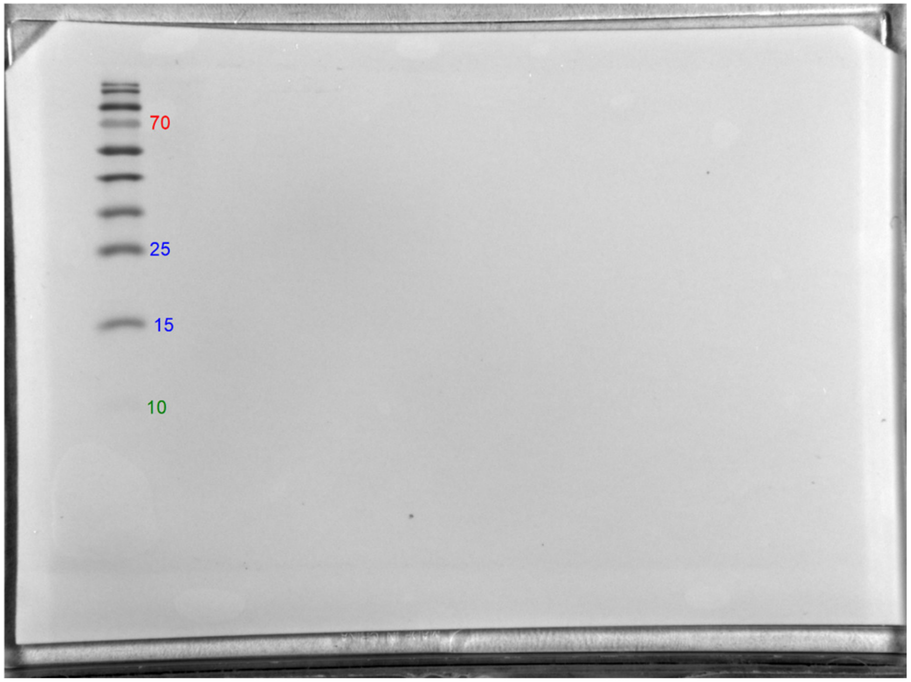

Section used for representative blot is boxed in red.
This blot was probed first for 4HNE and then reprobed for GPx4.

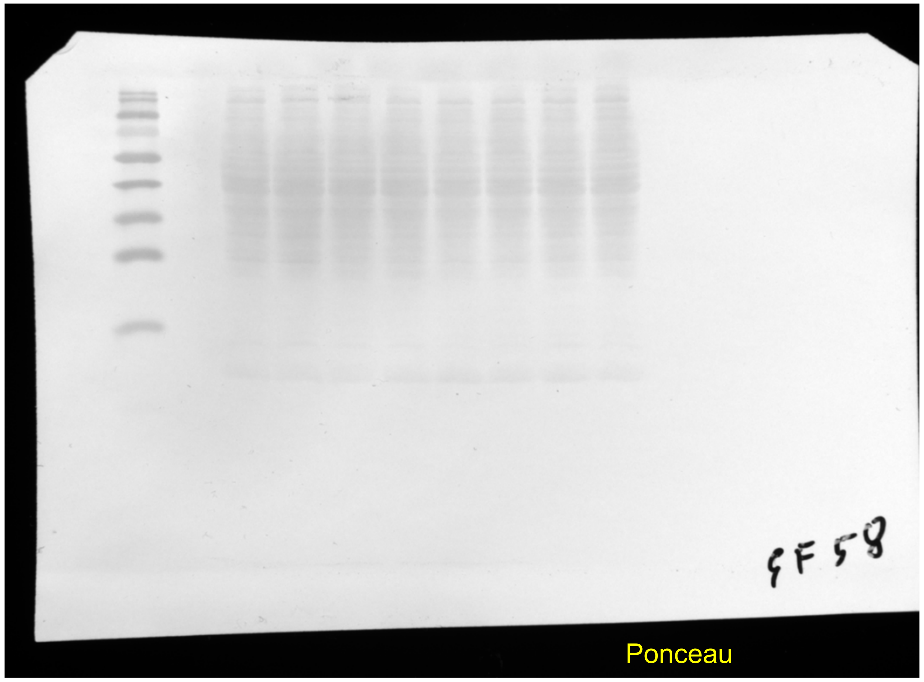

Confirmatory blot for GPx4 showing a single band.

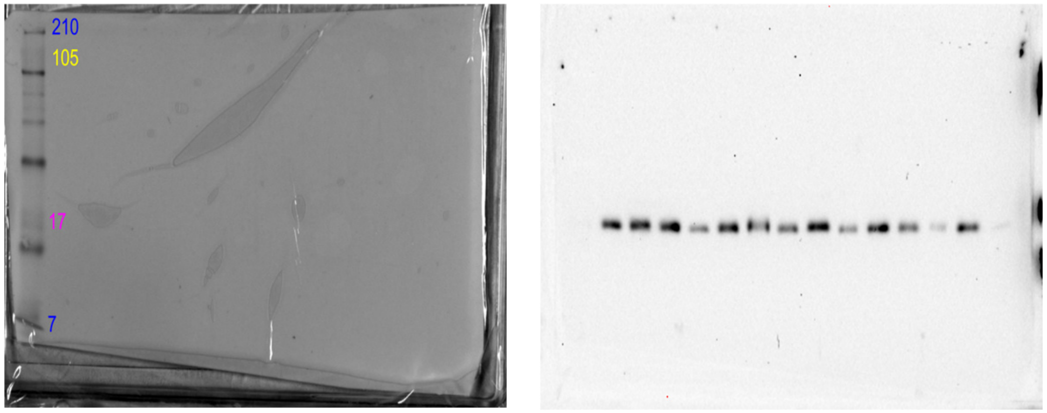

Complex IV (Comp IV)

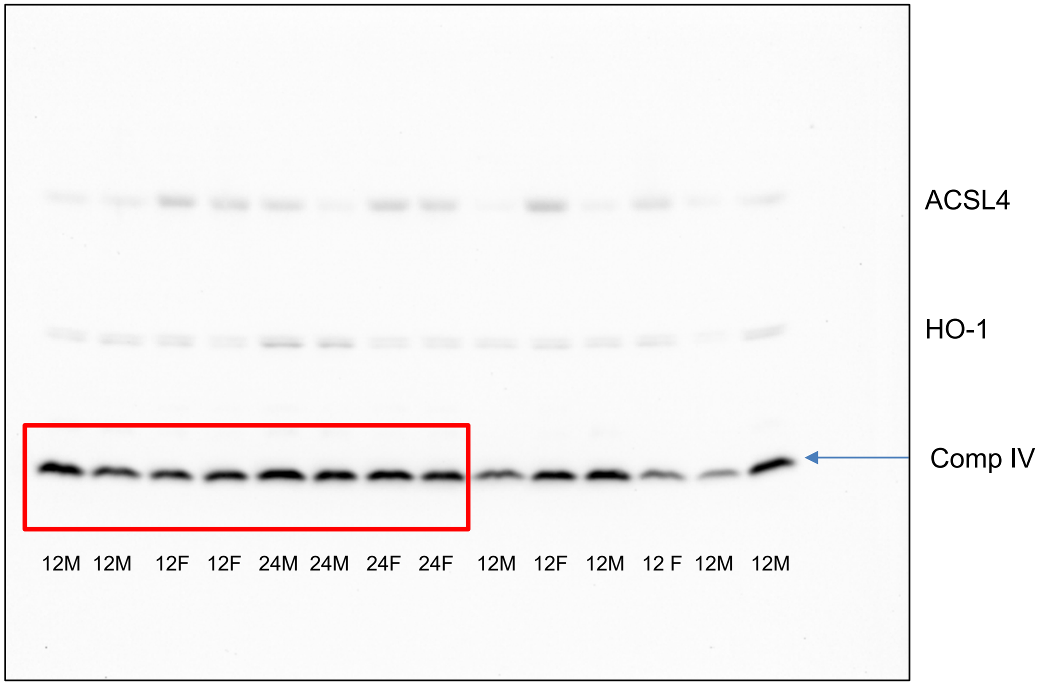

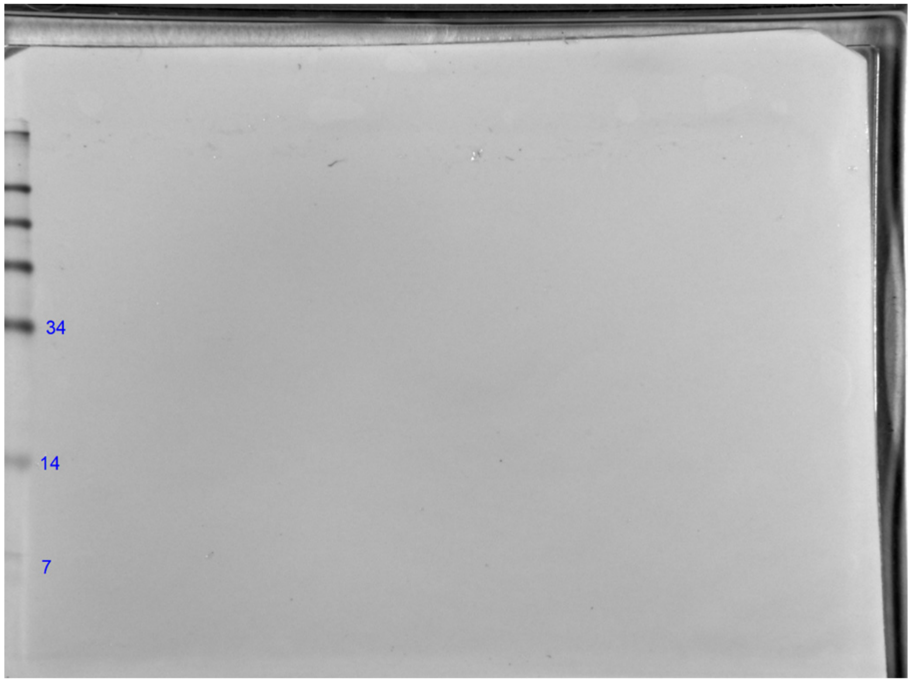

Section used for representative blot is boxed in red.
This blot was probed first for ACSL4 and HO-1 and then reprobed for Comp IV.

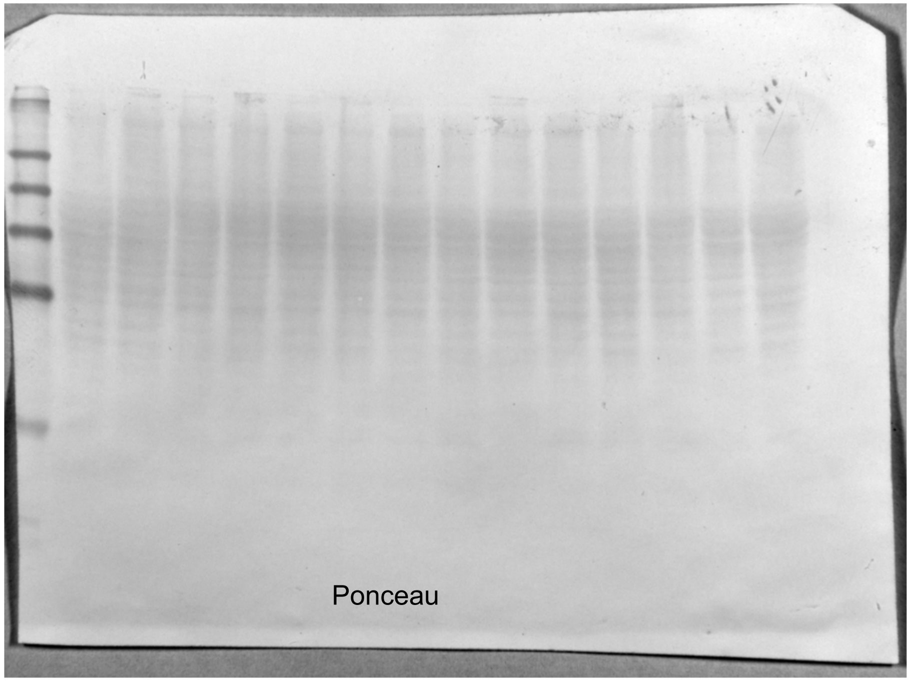

Manganese superoxide dismutase (MnSOD)

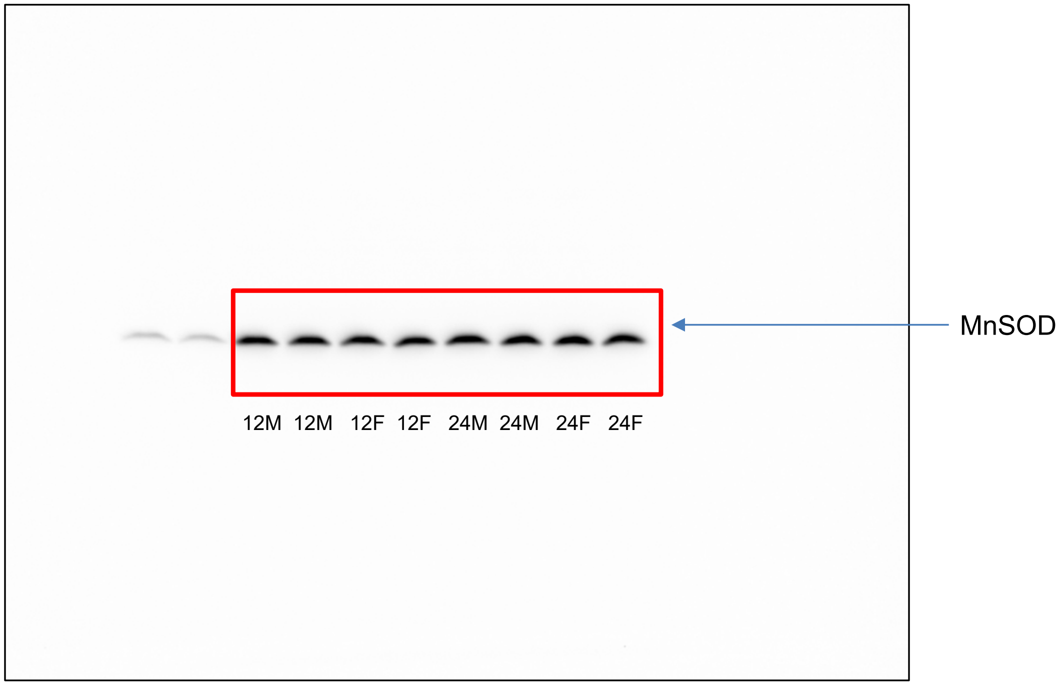

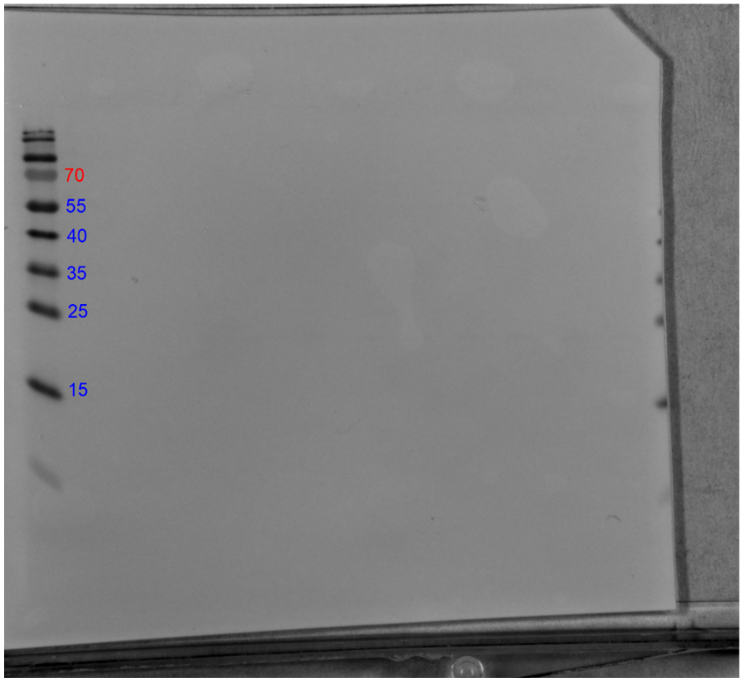

Brightfield

Section used for representative blot is boxed in red.
The first two samples are from spleen, which the Human Protein Atlas demonstrated was positive for ALOX-15.
This blot was probed first for ALOX15, and then for MnSOD. We did not detect ALOX15.

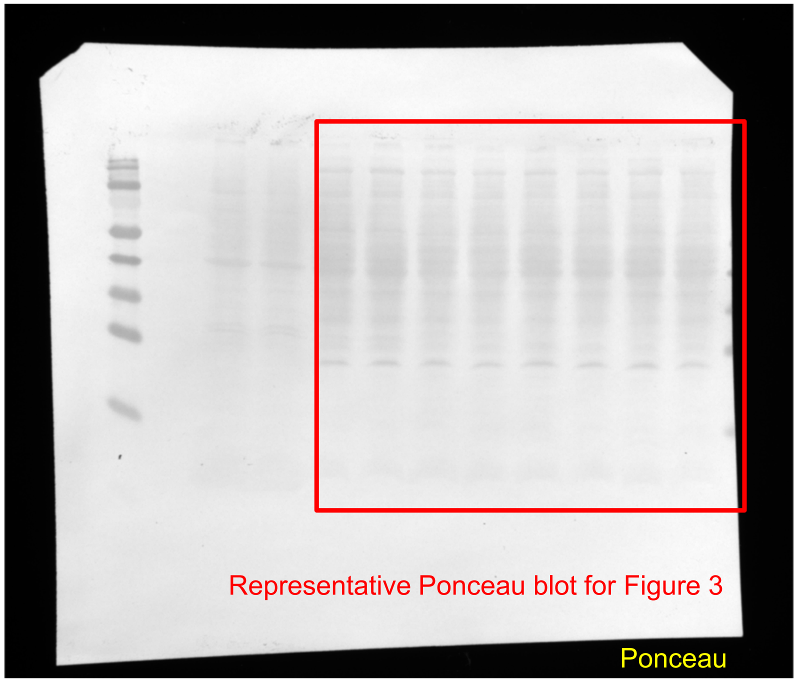

Thioredoxin-2 (Trx2)

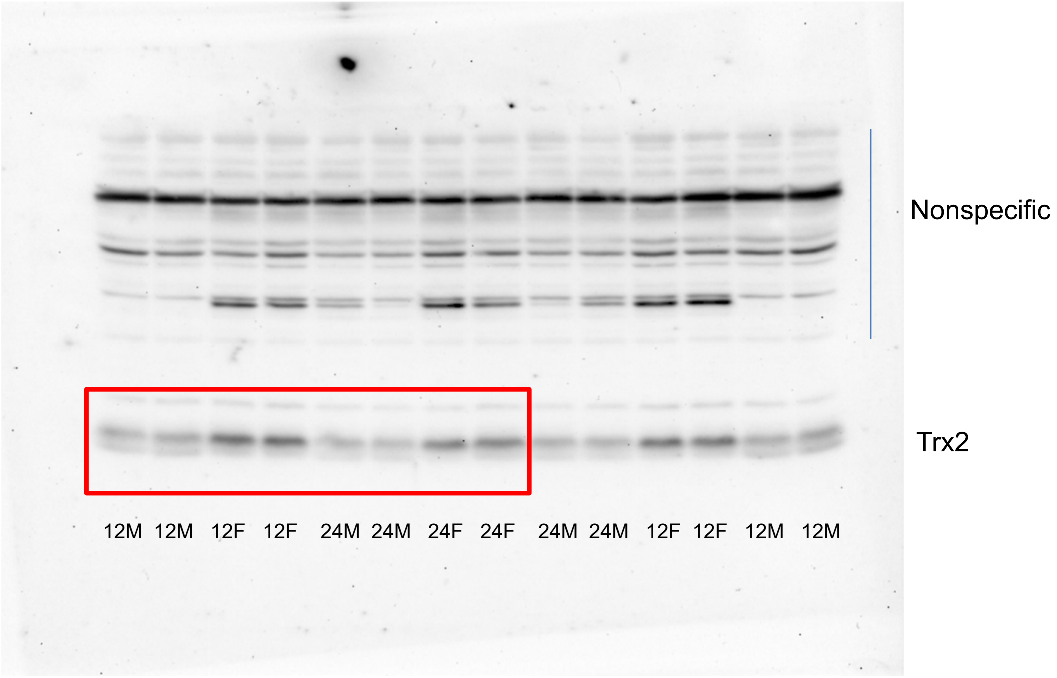

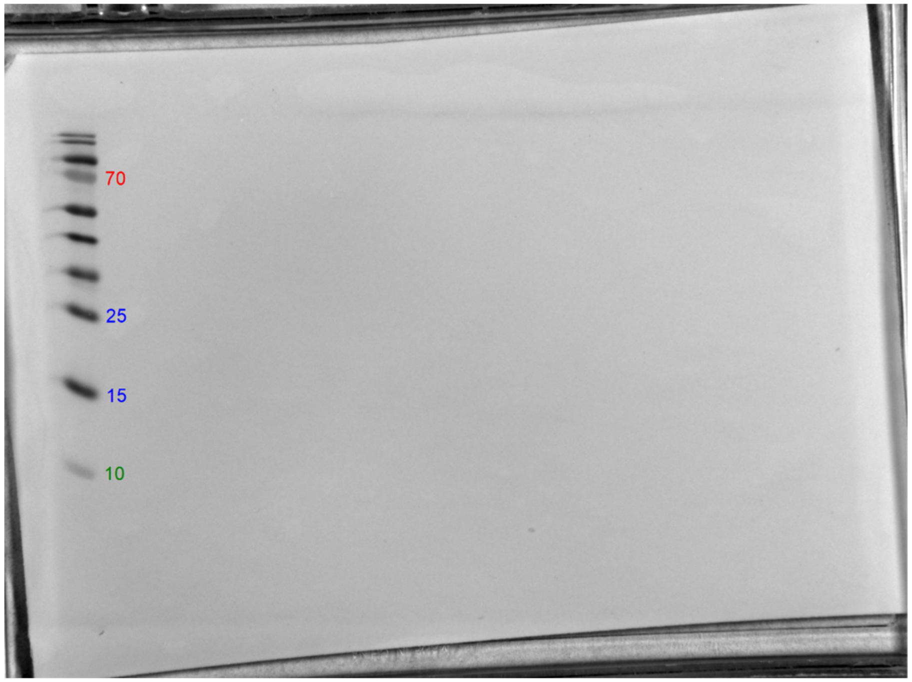

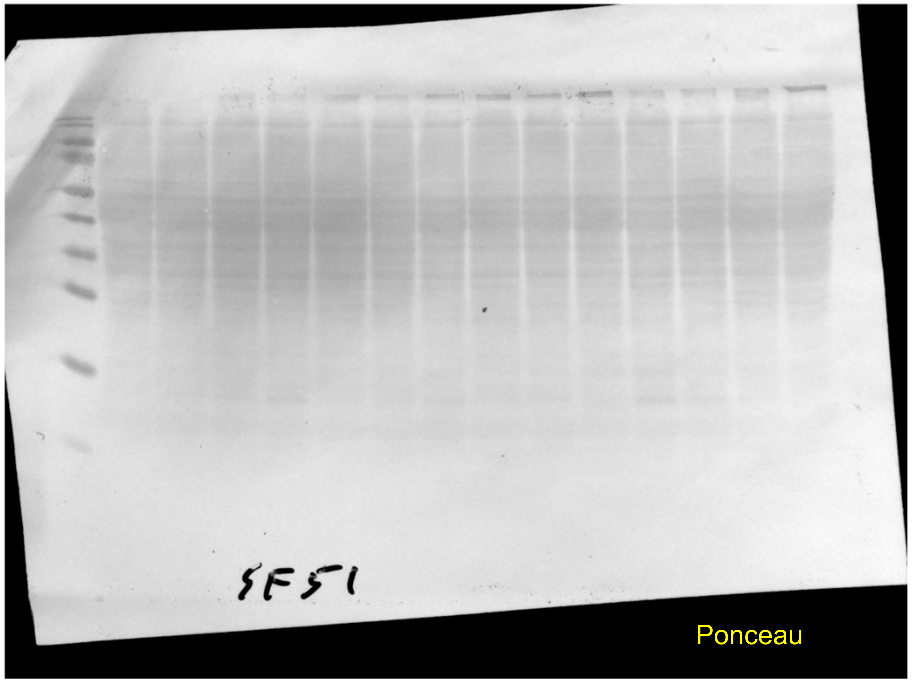

Cytosolic (Cyto) fractions and mitochondrial (Mito) fractions from a previous study probed for Trx2.

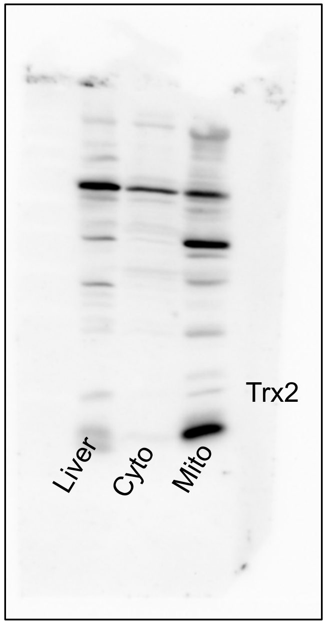

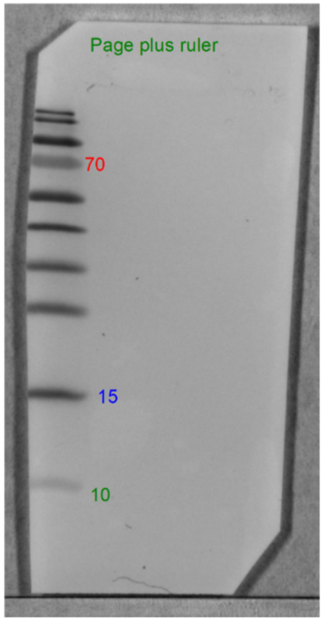

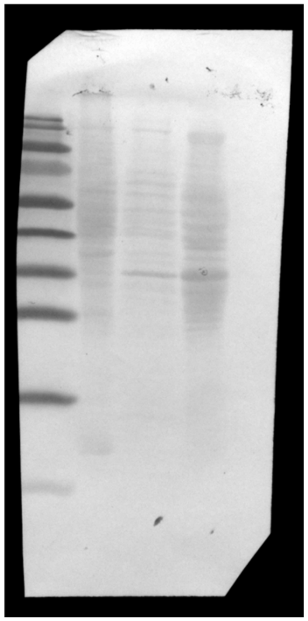

4-hydroxynonenal-modified proteins (4-HNE)

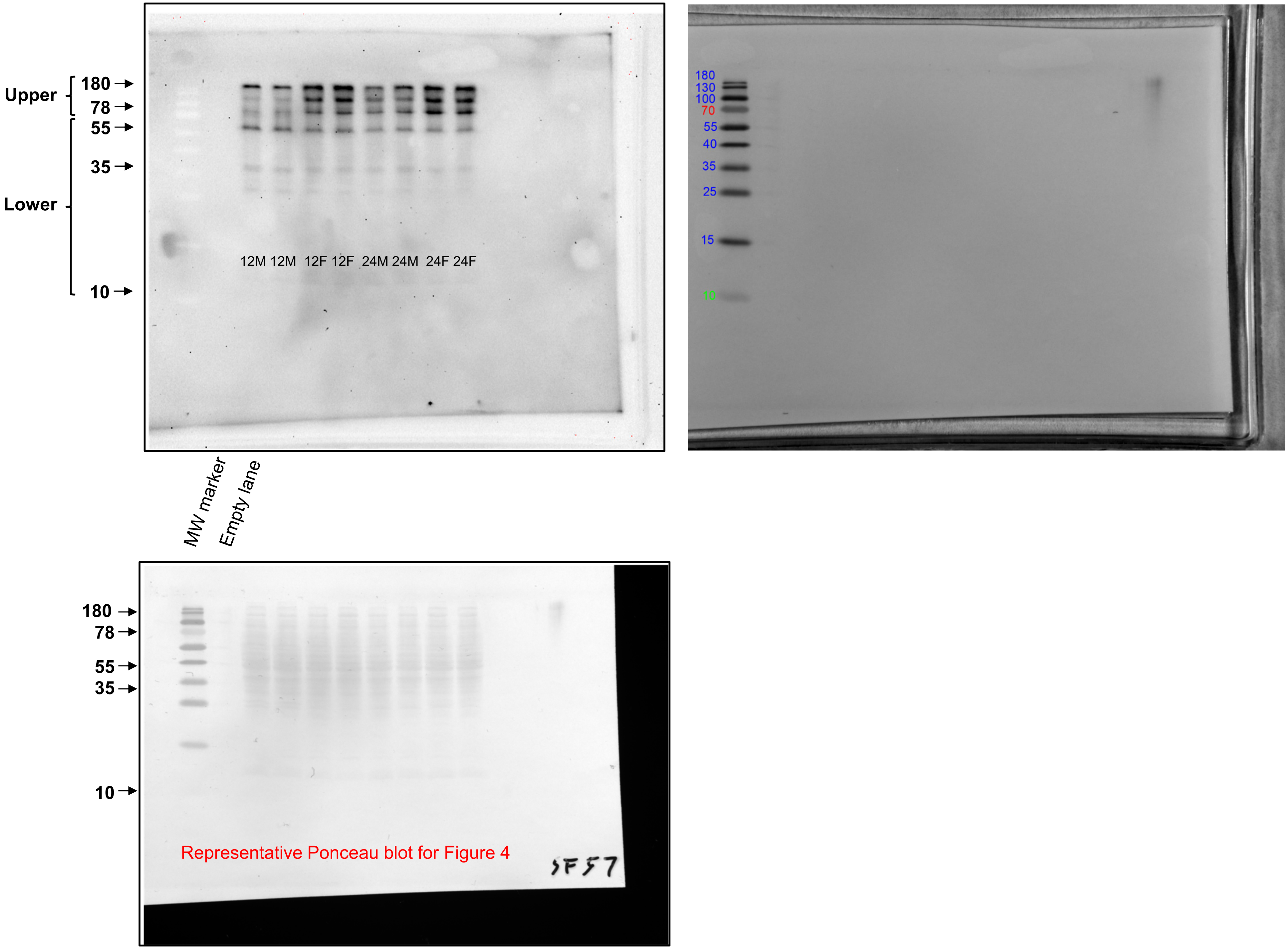

Supplement: Supplementary file 1 — Supporting Information [file EPH-109-2046-s001.pdf]
